# Measuring Indoor Air Quality and Engaging California Indian Stakeholders at the Win-River Resort and Casino: Collaborative Smoke-Free Policy Development

**DOI:** 10.3390/ijerph13010143

**Published:** 2016-01-20

**Authors:** Neil E. Klepeis, Narinder Dhaliwal, Gary Hayward, Viviana Acevedo-Bolton, Wayne R. Ott, Nathan Read, Steve Layton, Ruoting Jiang, Kai-Chung Cheng, Lynn M. Hildemann, James L. Repace, Stephanie Taylor, Seow-Ling Ong, Francisco O. Buchting, Juliet P. Lee, Roland S. Moore

**Affiliations:** 1 Education, Training, and Research, Inc., Scotts Valley, CA 95066, USA; narinderd@etr.org (N.D.); seowling@etr.org (S.-L.O.); 2 Department of Civil and Environmental Engineering, Stanford University, Stanford, CA 94305, USA; viviana.ab@gmail.com (V.A.-B.); wott1@stanford.edu (W.R.O.); ruotingever@gmail.com (R.J.); kccheng@stanford.edu (K.-C.C.); hildemann@stanford.edu (L.M.H.); 3 Neil Klepeis and Associates, Environmental Health Research and Consulting, Aromas, CA 95004, USA; 4 Win-River Resort & Casino, Redding Rancheria, Redding, CA 96001, USA; Gary.Hayward@win-river.com; 5 Shasta County Public Health Tobacco Education Program, Shasta County Public Health, Redding, CA 96001, USA; nread@co.shasta.ca.us (N.R.); layton12@gmail.com (S.L.); smtaylor@co.shasta.ca.us (S.T.); 6 Repace Associates, Inc., Secondhand Smoke Consultants, Bowie, MD 20720, USA; repace1@verizon.net; 7 Buchting Consulting, Oakland, CA 94612, USA; franciscobuchting@outlook.com; 8 Prevention Research Center, Pacific Institute for Research and Evaluation (PIRE), Oakland, CA 94612, USA; jlee@PREV.org (J.P.L.); roland@PREV.org (R.S.M.)

**Keywords:** air quality monitoring, smoke-free gambling, Native Americans, American Indians, hospitality business, worker protection policy, occupational exposure reduction, smoking, airborne nicotine, urinary cotinine, PM_2.5_, secondhand tobacco smoke

## Abstract

Most casinos owned by sovereign American Indian nations allow smoking, even in U.S. states such as California where state laws restrict workplace smoking. Collaborations between casinos and public health workers are needed to promote smoke-free policies that protect workers and patrons from secondhand tobacco smoke (SHS) exposure and risks. Over seven years, a coalition of public health professionals provided technical assistance to the Redding Rancheria tribe in Redding, California in establishing a smoke-free policy at the Win-River Resort and Casino. The coalition provided information to the casino general manager that included site-specific measurement of employee and visitor PM_2.5_ personal exposure, area concentrations of airborne nicotine and PM_2.5_, visitor urinary cotinine, and patron and staff opinions (surveys, focus groups, and a Town Hall meeting). The manager communicated results to tribal membership, including evidence of high SHS exposures and support for a smoke-free policy. Subsequently, in concert with hotel expansion, the Redding Rancheria Tribal Council voted to accept a 100% restriction of smoking inside the casino, whereupon PM_2.5_ exposure in main smoking areas dropped by 98%. A 70% partial-smoke-free policy was instituted ~1 year later in the face of revenue loss. The success of the collaboration in promoting a smoke-free policy, and the key element of air quality feedback, which appeared to be a central driver, may provide a model for similar efforts.

## 1. Introduction

The 1987 Cabazon U.S. Supreme Court ruling and subsequent Indian Gaming Regulatory Act (IGRA) of 1988 acknowledged that sovereign tribes recognized by the U.S. federal government may enter into compacts with states to establish casinos [1]. In their compact negotiations, tribes are not necessarily required to comply with state laws, including smoke-free workplace laws. In the past two decades, in which California Indian tribes have exercised their rights to conduct gaming operations, California has seen a major increase in the number of casinos that allow smoking. There are currently 69 tribal-owned casinos in California operating more than 70,000 slot machines, with annual revenues of $7 billion [2,3]. Nearly all of these casinos now allow indoor smoking.

Despite the large number of casinos permitting indoor smoking in California and elsewhere, relatively few published data are available on casino air quality and personal exposures of casino visitors and staff to the airborne pollutants present in secondhand tobacco smoke (SHS), including PM_2.5_ (fine airborne particles). Casinos are unique indoor environments that are typically much larger than traditional pubs and restaurants, have more complex building layouts and ventilation systems, and, during peak hours, contain very large numbers of patrons, of whom roughly 5% to 20% are actively-smoking at a time [4,5,6,7]. 

Recent evidence shows that PM_2.5_ levels can be high in casinos that allow smoking [5,6,7] and that casino workers are being exposed to hazardous levels of toxic substances present in SHS [8]. Babb *et al.* [9] conducted an extensive review of the published peer-reviewed literature on SHS in casinos. This review included studies of casino air quality (PM_2.5_ and airborne nicotine), biomarkers of exposure (e.g., cotinine), health outcomes, smoking prevalence of casino patrons and problem gamblers, and the economic impact of smoke-free policies. They concluded that the exposure of employees and patrons to SHS in casinos poses a significant, preventable risk to health.

The biomarker cotinine (e.g., in serum, urine or saliva) and airborne nicotine are commonly used as tobacco-specific indicators of SHS exposure, with PM_2.5_ providing a non-specific indicator with high SHS sensitivity [10,11]. Apart from its use as an SHS tracer, the U.S. Environmental Protection Agency (U.S. EPA) has promulgated a PM_2.5_ National Ambient Air Quality Standard of 35 μg/m^3^ (micrograms per cubic meter) averaged over 24 h that applies to outdoor air nationwide and is designed to protect public health [12]. Although the composition of SHS particles differs from outdoor ambient particles, Pope *et al.* [13] reported that PM_2.5_ in outdoor air and PM_2.5_ from SHS have similar toxicity. Fine particles can be inhaled deep into the lungs, and PM_2.5_ from SHS is associated with lung disease, decreased lung function, asthma attacks, heart attacks, and cardiac arrhythmias [14].

Brief SHS exposures have been associated with adverse respiratory and cardiovascular health [15]. Pope *et al.* [16] reported that exposure to 53 μg/m^3^ of PM_2.5_ from SHS for 1.75 h caused a decrease in the heart rate variability in human subjects of 2.3% for each 10 μg/m^3^ increase in PM_2.5_. Barnoya and Glantz ([17], p. 2684) state: “In many cases, the effects of even brief (minutes or hours) passive smoking are nearly as large as those from chronic active smoking”. Their review of the cardiovascular effects of SHS indicates that increases in aortic stiffness were observed after only 4 min of exposure to SHS, and exposure for 20 min was associated with activation of blood platelets, which could damage the lining of arteries and facilitate atherosclerosis. A number of other researchers have reported adverse physiological effects associated with short-term exposure to SHS, such as damage to the endothelium, the first inner layer of the arteries that is in contact with the blood [18,19,20].

While relatively few smoke-free casinos exist in the U.S. and the world, the Redding Rancheria, the tribal sovereign nation of California Indians located in Redding, CA (USA) that owns and oversees the Win-River Resort and Casino, expressed an early interest in becoming smoke-free in light of the adverse health effects associated with SHS. The Win-River casino has a total smoking and nonsmoking gambling floor area of approximately 24,000 square feet containing approximately 750 slot machines. In 2008, the Win-River Casino General Manager, Mr. Gary Hayward (a tribal member), sought technical assistance and information regarding the health effects of SHS in the casino, particularly as it affected the casino’s workforce. Mr. Hayward contacted the Shasta County Tobacco Education Program (SCTEP), located in Redding, CA, and the California Clean Air Project (CCAP), a state-funded public health initiative located in Sacramento, CA (USA), which promotes smoke-free tribal casinos. As a result of this contact, a Casino Advisory Committee (CAC) was formed, which would provide materials and support to Mr. Hayward as he brought information back to the tribal leadership, facilitated by a longstanding, trusting relationship between the casino general manager and the Tribal Council, on which he had served. As part of this process, the CAC was invited by the Win-River management to perform opinion surveys of patrons and employees and to perform an indoor air study. Both of these data collection efforts would provide site-specific educational materials to the casino, with the intent of characterizing potential SHS exposures in the casino building and the perceived benefits and barriers to a smoke-free policy. The casino management provided the CAC with free access to all areas of the casino for monitoring and person-counting activities, offering a unique opportunity to measure air quality levels in a normally-operating casino. Although progress toward casino-wide smoking restrictions suffered a setback in 2009 due to the United States’ financial downturn, CCAP maintained a relationship with Gary Hayward and the Win-River casino, carrying out additional air monitoring, surveys, focus groups, and a Town Hall-style meeting. The Tribal Council, after conferring with the membership of the tribe, ultimately voted to adopt a 100% smoke-free policy in early 2014.

This paper describes the detailed methods, history, and outcomes of an exploratory effort to promote a smoke-free casino using a collaborative approach in which a technical advisory committee assists casino staff in communicating information to tribal membership. The primary hypothesis, which drove this exploration, is that clear knowledge of SHS-contaminated air quality in the casino and accompanying health risks, when communicated to tribal membership and leadership, would be a major factor in moving a 100% smoke-free policy forward. We also hypothesized that concerns about air quality and health would be moderated by financial concerns, especially the loss of revenue and income for employees and tribal members. With this work, we seek to explore and evaluate the actual impact of air quality feedback and opinion surveys, identify specific barriers to smoke-free policy adoption, and disseminate information that may motivate other casinos to take measures to protect the health of patrons and employees. Our approach may provide valuable background data, templates, or guidelines for other researchers, public health workers, or casino managers considering or studying protective smoking policies. Furthermore, the scientific and public health benefits to reporting on before-and-after SHS concentrations in the casino include an improved understanding of the contribution of smoking to indoor air pollution in gaming and hospitality establishments.

Win-River is among the first few tribal casinos to have voluntarily adopted a smoke-free policy; a smaller Indian casino located in Northern California (Lucky Bear) has continuously been smoke-free [5], and several casinos in New Mexico have established 100% smoke-free policies as well. A subset of other U.S. casinos not owned by tribal entities have involuntarily become smoke-free due to state or local laws [21,22]. 

## 2. Methods

The Win-River casino was first established as a bingo hall in 1993. Smoke-free policy change efforts at Win-River originated in 1999 when the Redding Rancheria was contacted by Shasta County Public Health Department, which helped the casino management in their decision to establish a smoke-free area in the casino. A bid failed at this time for a casino-wide smoking restriction. No efforts to explore a smoke-free policy occurred from 1999 until May 2008, when General Manager Gary Hayward contacted the Shasta County Tobacco Education Program (SCTEP) for technical assistance and SHS information. In the same month, a Casino Advisory Committee (CAC) was formed to assist Redding Rancheria, consisting of SCTEP, the California Clean Air Project (CCAP), and Dr. Neil Klepeis, an Environmental Health and SHS research scientist. A tabulated historical time-line of the collaborative’s activities to lay the groundwork for establishing a smoke-free policy at the Win-River Resort and Casino is included in the Supplementary Information (Table S1).

An integral part of the assistance provided to Redding Rancheria was to perform scientific air quality testing for the purpose of informing the tribal membership about SHS exposures and health risks occurring in the casino. Initial air testing took place in May 2008 with subsequent tests in June and October of the same year. In June and July 2008, opinion survey forms were mailed to 1000 patrons on the adoption of smoke-free policies. Casino and Rancheria employees and the membership were also surveyed. The air testing and survey results were presented to the Tribal Council, which has governing power and consists of seven members with three alternates, and General Membership (~350 members) in August 2008, at which time the membership voted to research the possible adoption of a smoke-free policy. Due to the nation’s financial downturn, the tribe decided to delay its smoke-free policy decision in February 2009. CCAP continued to be in contact with Redding Rancheria and Manager Hayward from 2010 to 2014 to maintain support of the smoke-free issue. Follow-up air testing was done in July 2011 with follow-up patron surveys in June 2012. Staff and community focus groups and a Town Hall meeting with casino patrons were conducted in August 2013. Follow-up data were presented to the Tribal Council on 23 January 2014, at which time they voted to implement the smoke-free policy. The casino-wide smoke-free policy was adopted on 14 March 2014, with post-policy air monitoring performed on 26 March 2014 and a post-policy employee survey in May–June 2014. In a more recent development, the Tribal Council voted in February 2015 to amend the smoke-free policy to permit smoking on 30% of the casino floor.

Below, we describe the methodology and rationale for the instruments and materials used to provide technical assistance to Redding Rancheria and tribal leadership in support of a smoke-free policy for the Win-River Casino. The results of all environmental monitoring and stakeholder-related activities (opinion surveys, focus groups, and Town Hall meeting) were also presented in reports to the Tribal Council. Evaluation reports on funded tobacco control activities related to the Win-River casino were provided to the state of California [23,24].

### 2.1. Air Quality Evaluation

The collection and reporting of air quality data to the Tribal Council and General Membership was designed to provide feedback on health risks for staff and patrons on the magnitude of secondhand tobacco smoke (SHS) exposure that could occur in the casino. Over a series of weekend visits, the CAC measured personal exposure air concentrations of PM_2.5_ on up to 4 visitors and 3–4 employees each day, airborne nicotine and/or PM_2.5_ in several fixed-site (area) locations, and counts of active smokers and total patrons. The CAC also measured urinary cotinine measures in 4 CAC visitors on one visit. In light of the Redding Rancheria's sensitivity to employee health and the documented acute and chronic health effects associated with SHS, the results of the air quality evaluation—provided as formal presentations or informal reports—were expected to provide key evidence for deliberations over the casino’s smoke-free policy. The materials consisted of short descriptions of the monitoring and counting methodology with one or more charts showing a typical person’s personal particle exposure profile (concentration versus time), and tabular data on mean and peak (maximum) air pollutant levels and cotinine levels (similar to material included in the Supplementary Information; Table S2, Table S3, Table S4 and Table S5). The reports and presentations illustrated the high levels of SHS exposure that occurred in the casino in different locations with active smokers relative to the cleaner air observed in nonsmoking areas and the outdoors. Smoker counts provided valuable information on the proportion of people who smoke in the casino at a given time.

#### 2.1.1. Casino Dimensions and Ventilation

**Figure 1:**
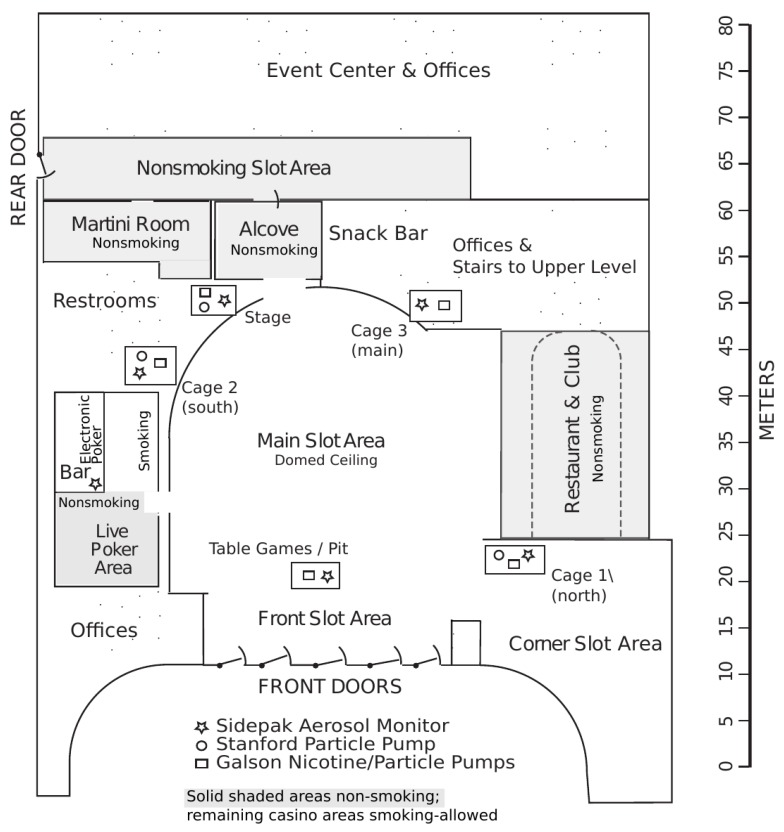
Schematic showing the casino layout and fixed-site air monitoring positions during the 2008 monitoring periods before the smoke-free policy was adopted. The layout was changed slightly in 2011 and further in 2014 subsequent to hotel construction, which was completed in December 2013. The poker area was moved to the martini bar area, and the corner slots area currently opens up to the hotel lobby.

As part of the air quality evaluation, the volume dimensions of the casino were measured by pacing along the walls of each room in the casino and using a sonic distance-measurement device (DM 550 Sonic Measure, Zircon Corp., Campbell, CA, USA). The central gambling area of the casino (smoking slots and tables, plus connected alcove and bar areas) had a rough interior volume of 9000 cubic meters (~320,000 ft^3^), a total floor area of approximately 1900 square meters (20,000 ft^2^), and ceiling heights up to 18 feet (see Figure 1). Ventilation is important in considerations of air quality. The casino’s Heating, Ventilation, and Air-Conditioning System (HVAC), as reported by the building engineer, was designed to deliver 168,000 cubic feet of air per minute at 100% capacity typically with 25% to 50% outside air. Thus, for capacities ranging from 50% to 100%, the system was expected to deliver between 21,000 and 84,000 cubic feet per minute of fresh air. With an approximate volume of 320,000 cubic feet, the expected air change rate would be 4 to 16 per hour. For comparison, residential air change rates have a central tendency of 0.5 air changes per hour [25]. The casino HVAC filtration system was reported to consist of 35%-efficiency 2-inch pleated filters, followed by electronic filtration, and finally by 12-inch 95% HEPA filters.

#### 2.1.2. Particle Measurement Methods

We performed intensive real-time monitoring of personal exposure and indoor fixed-site PM_2.5_ concentrations in the Win-River Casino and Resort on Friday and Saturday evenings during five separate 2-day visits (10 total days) in May, June and October 2008, December 2011, and March 2014 (see Figure 1 for fixed-site sampling locations). All data were provided to casino management and, subsequently, reported to tribal leadership. The fixed-site monitoring usually began in the mid-afternoon and lasted up to ~10 h per day. During each of the 10 days of monitoring at the casino, one to four CAC visitors wearing real-time SidePak™ AM510 Aerosol Monitors (TSI, Inc., Shoreview, MN, USA)—with inlets in their breathing zones—continuously-measured their PM_2.5_ personal exposure concentrations and/or performed counts of active smokers and total patrons in multiple casino locations, including smoking and nonsmoking areas, and the outdoors. When in gambling areas, they acted as typical patrons would, sitting at table and slot games. On the visits for Days 3–8 (6 total days), 3–4 Win-River casino employees wore SidePak monitors to measure their breathing-zone personal exposure. The personal monitoring and counting methods were identical to those used by Jiang *et al.* [5] and Klepeis *et al.* [7].

Before each use, the SidePak monitors were fitted with 2.5-micrometer size-selective impactor inlets and their flow rate was set at 1.7 L per minute (LPM). Each SidePak’s impactor was cleaned, and the instrument was zero-calibrated, prior to each use. We set a data-logging interval of 10 s or 1 min. We converted the instrument response to PM_2.5_ mass concentration using custom mass-calibration factors. As described by Jiang *et al.* [26], we performed both on-site and laboratory-based gravimetric calibration of the SidePak monitors used in the Win-River casino using filters and sampling pumps. For on-site measurements, we also placed gravimetric samplers throughout the casino during the six visits in 2008 (see Figure 1 for sampling locations). 

The overall mean on-site calibration factor was determined by Jiang *et al.* [26] to be 0.29. The laboratory SHS calibration factors, determined at two time points spaced approximately a year apart, were consistent with the on-site values ranging from 0.24 to 0.31. The mean calibration factor from laboratory experiments was nearly identical to the on-site casino value at 0.28 to 0.29, changing very little over the year between laboratory evaluations. In addition, Jiang *et al.* [26] determined the measurement precision of SidePak monitors, based on a collocated set of 17 SidePak units, to be 5%, indicating excellent consistency between individual units. The strong similarity between field and laboratory-based calibration factor determinations validates the use of the SidePak to measure PM_2.5_ mass levels in the smoking casino environment. For very low levels, e.g., in the absence of tobacco smoke, non-source-specific calibration factors introduce small absolute errors. For example, for a level of 5 μg/m^3^, an error in calibration factor of 50% would introduce absolute uncertainty of only 2.5 μg/m^3^ in the measurement.

#### 2.1.3. Nicotine and Cotinine Methods

We measured airborne nicotine at fixed-sites in the casino (see Figure 1) on 4 days (Days 3–6) in June/October 2008. We used a modified NIOSH Method 2551 with pumps and XAD-4 sorbent media and subsequent GC/NPD analysis performed by a commercial laboratory (Galson Labs, Buffalo, NY, USA). On a Saturday visit in June 2008, urine samples of 4 nonsmoking CAC members were taken before and after being exposed to SHS in the casino, and only in the casino, for times ranging from 1.5 to 10 h during which they acted as patrons (the Supplementary Information has a tabular summary; Table S2). The post-specimens were taken 45 min to 4 h after the last exposure occurred. The urine samples were analyzed for cotinine (metabolite of nicotine) to assess the SHS exposure level during the casino visit. The analysis was performed by Esther Giesbrecht, clinical lab manager at the Centre for Addiction and Mental Health (Toronto, ON, Canada), using GC analysis and normalized with respect to creatinine level. The samples were hydrolyzed to release bound cotinine and hydroxycotinine.

### 2.2. Stakeholder-Opinion Activities

**Figure 2:**
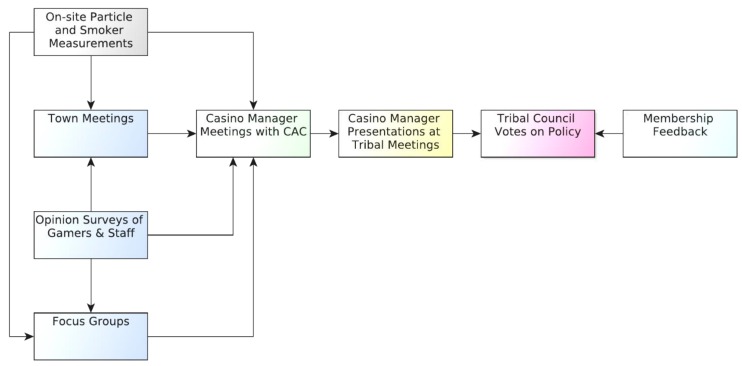
Diagram showing the linkages between material elements of the policy change support efforts and specific stakeholder sessions. CAC was the Casino Advisory Committee consisting of CCAP, SCTEP, and Dr. Neil Klepeis.

The CAC’s process included the collection of opinion data from Win-River Casino patrons, Win-River Casino employees, and Redding Rancheria employees. The Supplementary Information contains a tabulated history of activities and decisions by casino representatives related to the smoke-free policy (Table S1). The goals of the stakeholder activities were to: (1) Measure the level of support for a smoke-free casino by key community members, patrons and employees; (2) Identify barriers to the social and political acceptance of a smoke-free casino policy; (3) Gather perspectives on potential benefits of a smoke-free casino; and (4) Elicit patrons’ feedback on possible smoke-free implementation strategies. The CAC conducted the following four types of stakeholder-related activities: (1) Establishment of collegial relationships and agreements with the tribe and casino management; (2) Formal surveys of patrons, owners, and employees; (3) Administration of focus groups with patrons and community members who use casino facilities; and (4) Leading of a Town Hall meeting with patrons. In addition, the CAC was given access to data from annual organizational surveys performed by the casino and was allowed to perform key informant interviews with some Redding Rancheria staff. Figure 2 illustrates data categories and feedback linkages that were part of the CAC process. All data were summarized separately and shared with the casino General Manager, Mr. Gary Hayward, who presented them to the Tribal Council in support of a smoke-free casino policy. The presentation of the results of SHS environmental monitoring to all participants was a core element in focus groups, the Town Hall meeting, and interpersonal contacts.

#### 2.2.1. Direct Contacts with Casino Staff: 2008–2013

The CAC provided ongoing materials, including reports and presentation slides, and verbal technical assistance directly to Manager Hayward and an administrative assistant. Ms. Dhaliwal (CCAP project director) made frequent contact with Manager Hayward by email, phone, and in-person communication (~2 times a month on average). In-person meetings were held with Manager Hayward on a quarterly basis with follow-up contact via phone and email. These contacts were maintained to ensure that the issue did not get “left behind”—since business decisions consumed much of the General Manager’s and Tribal Council’s time. The discussions with Manager Hayward were focused on explaining the results of air monitoring and surveys and answering any questions. The discussions also included the general state of the casino and how it might affect the smoke-free policy adoption, such as the planned expansion project to include the hotel, changes in casino floor usage, the total number of machines on the floor throughout the years, the economic downturn and how it was affecting the casino revenue, ca. 2009–2012 (e.g., reduction in machines on the casino floor and the extension of pooled health cost self-insurance coverage of casino employees in 2012).

#### 2.2.2. Patron, Employee, and Tribal Member Surveys—2008

The objective of the first opinion surveys in June and July 2008 was to evaluate support for the policy to gauge potential success with continuing smoke-free efforts and provide motivating data to stakeholders. The survey respondents were not made aware of the results of air quality surveys. Four groups were chosen to be surveyed as stakeholders in any potential smoke-free policy: (1) Casino patrons; (2) Casino employees; (3) Rancheria employees; and (4) Rancheria members. All survey instruments were developed by CCAP, SCTEP, and by Manager Hayward [23] based on the Secondhand Smoke Casino Advocacy Guide from the California Rural Indian Health Board (https://www.crihb.org/) and also based on questions from SHS opinion surveys conducted and evaluated by CCAP’s statewide SHS Technical Assistance and Advocacy Project (funded by the California Tobacco Control Program starting in 2005) in relation to SHS exposure in businesses, multi-unit housing, outdoor dining, bus stops, *etc.* (both outdoors and indoors). CCAP derived some opinion questions from those in the California Department of Public Health’s Tobacco Control Evaluation Center’s (TCEC) database used to evaluate support and resistance to California’s smoke-free bar law [27].

One thousand casino patrons were randomly-selected from three tiers of gaming involvement (low-stakes to high-stakes) from the casino’s Players Club membership list by the casino management (Manager Hayward assisted by casino staff). The proportion of the sample across the three tiers was matched to the distribution of the Players Club tiers. Since the casino revenues are largely made up of income from frequent, but low-stakes “C”-tier visitors to the casino, the largest group sampled was from the “C” tier, whereas the smallest group sampled was from the “A” tier, representing the high-stakes players but less-frequent visitors to the casino. In June and July 2008, the casino mailed paper surveys to 1000 selected patrons along with a $10 “free play” incentive for completing the survey. 297 surveys were returned, for a patron response rate of 29.7% (respondent proportions by tier: 6.4% Tier A; 28.8% Tier B; 64.8% Tier C). Respondents were asked about their current smoking status, how frequently they visited the casino, how frequently they would visit if there were a smoke-free policy, how frequently they would visit if there were designated outdoor smoking areas, and what they like best about the casino. Additionally, non-smokers were asked whether they were bothered by smoke in the casino, whereas smokers were asked whether they smoke while they play.

All casino employees were given a chance to respond to the survey, which was emailed to them with a link to a SurveyMonkey web-based survey (https://www.surveymonkey.com/). Of the 366 casino employees, 153 (41.8%) responded to the survey. Staff survey questions included current smoking status, whether respondents were bothered (adversely affected in any subjective way) by SHS in their work area, whether they would prefer to work in a nonsmoking room in the casino, and whether they would prefer to work in a smoke-free casino.

All Rancheria employees and Rancheria members were given a chance to respond to the survey, which was identical for the two groups. Rancheria employees were emailed a link to a SurveyMonkey survey. Paper surveys were distributed to Rancheria members. Fifty-seven (41.9%) of the 136 Rancheria employees responded to the survey, while 12 (10%) of the 120 Rancheria members returned surveys. Questions included respondents’ current smoking status, whether respondents were concerned that casino employees are breathing SHS, whether they would prefer that casino employees work in a smoke-free casino, and whether they would support a smoke-free policy in the casino. 

Data from paper surveys from casino patrons and Rancheria members were entered by SCTEP staff into SurveyMonkey website forms developed for data entry. Responses from employees of the casino and Rancheria were entered by each respondent into SurveyMonkey when completing the survey. Basic frequencies were calculated by SCTEP for all surveys using the SurveyMonkey reporting feature, and data for all surveys were downloaded into raw data files for importation into the Statistical Package for the Social Sciences (SPSS) [28] for data cleaning and cross-tabulation analysis.

#### 2.2.3. Employee and Patron Follow-Up Surveys—2012

In 2012, four years after the initial survey, similarly-worded follow-up surveys were conducted with Win-River patrons and employees. All the questions were aimed at the same core concepts of participant perceptions of harm, value, inconvenience, cost, and benefit of smoke-free policy expansion. Wording was slightly altered to fit a particular role (e.g., casino employee *vs.* occasional patron). A total of 1990 postcards were mailed to patrons randomly-selected from three tiers of the Players Club membership list by the casino, as noted above, again in exchange for a $10 free play incentive. In all, 1250 surveys were completed, for a response rate of 63%. For casino employees, all staff members were given a chance to respond to the survey in the form of an email with a link to an online SurveyMonkey survey. A total of 97 employee surveys were completed, for a response rate of 32%. 

#### 2.2.4. Focus Groups Concerning Smoke-Free Gaming—2013

Three focus groups were conducted by CCAP staff in August 2013, comprised of two groups of Win-River Casino patrons, and one group of local lead agency staff and members of the community who regularly utilize casino facilities (e.g., conference rooms or restaurants). The patrons were selected by the casino staff and invited to participate in the focus groups. A $25 gift-card incentive was provided to participants by CCAP. Participants in the patron focus groups were asked to brainstorm a list of potential benefits and barriers if a smoke-free policy were implemented at Win-River Casino. Respondents’ responses were recorded on flip chart paper, which were content-analyzed and reported by themes. Each focus group consisted of six respondents and lasted an average of 25 min. The focus groups were intended to help us better understand the range and nature of the diverse views surrounding the implementation of smoke-free policies in the casino. Since the number of focus groups was small, the analyses consisted of simple thematic analysis in which recurring kinds of responses were highlighted, along with dissenting views, in order to summarize the discussion. The focus group format was based on guidelines set forth in Greenbaum [29].

#### 2.2.5. Town Hall Meeting—2013

At the request of Win-River casino, CCAP conducted a Town Hall meeting concerning the potential reactions to establishing a 100% smoke-free policy with 23 patrons in August, 2013. Approximately 25 patrons were invited to participate; each received a $50 free play token if they attended the meeting, and if they brought a friend, they would both receive a $25 free play token. The selection was based on a distribution of patrons’ smoking behavior and their willingness to participate. Prior to the meeting, Win-River Casino had also elicited written feedback on the proposed policy from patrons visiting the casino. A total of 120 feedback cards were completed.

#### 2.2.6. Post-Policy Employee Survey—2014

The Win-River Resort & Casino performs an annual Organizational Climate Survey to assess employee characteristics and feedback. The May–June 2014 version of the survey, which obtained responses ~2–3 months after a casino-wide smoke-free policy went into effect, contained 12 questions asking about employee job type, gender, age, length of employment, smoking status, whether the employee had worked in a nonsmoking area prior to the smoke-free policy, their shift duration, whether they have a smoke-free home environment, their likelihood of entering a smoking cessation program or quitting smoking after the smoke-free policy, improvements to their health, any improvements they saw in the work following the new policy, and general comments about the policy. Of ~400 employees, 241 returned completed surveys (response rate of ~60%).

#### 2.2.7. Key Informant Interviews—2015

An understanding of the decision-making process of the Reading Rancheria Tribal Council was important to addressing our hypothesis and understanding the factors leading to smoke-free policies. To protect privacy and control access to sensitive or confidential tribal information, the CAC and Redding Rancheria tribe agreed to channel all communications through General Manager Hayward. Thus, Hayward mediated all informational contact between the CAC and the Tribal Council. He provided insight into the opinions of Tribal Council members in a formal key-informant interview performed in May 2015. The interview, performed by CCAP, included questions on the perceived reasons for the original 100% smoke-free vote, the subsequent roll-back to a partially-smoke-free casino, and open-ended discussion on lessons-learned and recommendations for future smoke-free efforts. In addition, in February 2015 CCAP performed a formal key-informant interview with a former alternate member of the Tribal Council, who was an alternate during decision-making regarding smoke-free policies.

### 2.3. Ethics

The field work described in this article was performed as a non-research, programmatic health-promotion effort funded by the California Department of Public Health Tobacco Control Program from 2007–2014. Subsequently, we analyzed the data under a 2011–2015 research grant from the state of California’s Tobacco Related Disease Research Program (TRDRP) to the Pacific Institute for Research and Evaluation (PIRE) with continuing approval from the PIRE Institutional Review Board (IRB). The IRB’s latest relevant approval was effective from 13 February 2015 to 13 February 2016: “Pursuant to 45 CFR 46, Pacific Institute’s PIRE IRB #1 has approved your submission. This project has been determined to be a Minimal Risk project.”

## 3. Results and Discussion

The results of air quality surveys showed that smoking caused high levels of particulate matter in the casino due to a small percentage of actively-smoking patrons. These results were conveyed to the casino membership, who, based primarily on this information, as well as secondarily from results of opinion surveys, ultimately decided to move towards a 100% smoke-free casino. Details from the results of the air quality (Section 3.1) and other surveys (Section 3.2) are presented and discussed below to document the type of feedback provided to the casino, and the character of particle levels in a typical casino, both before and after a 100% smoke-free policy. These details are of direct use by public health workers in other smoke-free casino efforts and as archival feedback for future work at Win-River. In Section 3.3, we describe results from the process of presenting data to Tribal Council, including results of a key informant interview with Mr. Hayward, the Casino Manager, and a former alternate member, in which they discussed reasons for the initial 100% smoke-free policy and the eventual roll-back to a partially-smoke-free policy. It was not possible to directly interview acting, individual Tribal Council members due to confidentiality agreements.

### 3.1. Air Quality Evaluation Results

#### 3.1.1. Counts of Total Patrons and Active Smokers

We obtained one to nine sets of complete count data for seven out of 10 Friday and Saturday visits between 8 PM and 12:00 PM in the main indoor smoking areas (slot and table game areas only, excluding designated nonsmoking areas and the bar, which had both a smoking and nonsmoking section). The count data consisted of the number of total and actively-smoking patrons. We found tight ranges of 391–454 for total patrons and 30–54 for active smokers (overall averages across the individual counts on each visit), and a narrow range of 8%–12% for the percentage of patrons (100× average active-smokers/average total patrons) who were observed smoking in the slot and table areas on each visit. These results are similar to those found in California casinos by Jiang *et al.* [5] and Klepeis *et al.* [7], who reported active-smoker percentage ranges of 5%–25% and 5%–10% from casino-to-casino, respectively (with overall average values of 11% and 7%).

#### 3.1.2. Cotinine and Nicotine Levels

A significant rise in urinary cotinine (cotinine + hydroxycotinine) was detected in the nonsmoking CAC visitors following their time spent in the casino (see Supplementary Information for details; Table S2). Cotinine levels measured after spending time in the casino ranged from 2.5–9.5 µg/L, a change of 2.2–9.3 µg/L, depending on the subject. These results are similar to an average increase in free cotinine of 1.9 µg/L (*i.e.*, 7.9 ng/mL in cotinine + hydroxycotinine) reported by Repace [30] for eight nonsmoking patrons each exposed to SHS over 4–5 h in Pennsylvania casinos. Since cotinine is a metabolite of nicotine, a unique constituent of tobacco smoke, these elevated levels of cotinine confirm that significant exposure to SHS occurred in the Win-River casino. Similarly, high levels of airborne nicotine, a unique tobacco-specific tracer, were also observed on the casino floor ranging from 3–10 µg/m^3^ (see Supplementary Information; Table S3), which is similar to area-wide airborne nicotine geometric-mean levels of 5–10 µg/m^3^ found by Achutan *et al.* [8] for three Las Vegas, Nevada, casinos and average values of 6–16 µg/m^3^ reported by Trout *et al.* [31] in a single casino. Our airborne nicotine results confirm that high levels of SHS were present in the Win-River casino air.

#### 3.1.3. Particle Levels

The mean levels at the Stage fixed-site location before the 100% smoke-free policy, which ranged from 24 to 49 µg/m^3^, were generally lower than those of other locations (see Figure 1 and Supplement Table S3). Note that the Supplement contains detailed tabular summaries of all the SidePak-derived mean and 1-min maximum PM_2.5_ personal exposure and fixed-site concentrations measured at Win-River casino (Table S3, Table S4 and Table S5). The mean concentrations in the Pit ranged from 60 to 87 µg/m^3^ and they were 34 to 74 µg/m^3^ in the Cage location. Similarly, the maximum 1-min concentrations in the Pit and Cage locations sometimes reached 200 to 300 µg/m^3^. Achutan *et al.* [8] found similar average area concentrations of 23 to 86 µg/m^3^ (respirable suspended particles) and Trout *et al.* [31] found comparable averages ranging from limit-of-detection (20–30 µg/m^3^) to 90 µg/m^3^. After the 100% smoke-free policy at Win-River went into effect, the fixed-site levels in the Pit on Days 9 & 10 dropped to 1–2 µg/m^3^, a decrease of 97% to 99% relative to Days 7 and 8. Clearly, the policy eliminated essentially all of the PM_2.5_ inside the casino, although outdoor fixed-site mean levels on Day 10 doubled relative to Day 5 and 6 levels, and the 1-min maximum increased by 6 times, likely due to outdoor smoking activity on the balcony near the monitors. The high outdoor levels on Days 3 and 4 were due to wildfire activity (forest fires) in the Redding, CA, area, but these do not appear to have affected indoor levels, since indoor concentrations were similar on Days 5 and 6 when no wildfires were present.

We found the Win-River casino employee personal PM_2.5_ exposures were consistent with measurements at the indoor fixed sites, with mean shift personal exposures ranging from 28 to 71 µg/m^3^ and maximum 1-min exposures ranging from 99 to 701 µg/m^3^ (see Supplementary Information for details; Table S4). Consistently-high maximum personal exposures can be explained by the close proximity between workers and smokers as they fulfill their duties (e.g., cashier and security guards approaching smoking customers or pit boss approaching tables with 1 or more smokers).

Visitors who acted as patrons (sitting at slot or tables games) had mean personal PM_2.5_ exposures in smoking slots areas that ranged from 53 to 82 µg/m^3^ (grouped data by day and location; see Supplementary Information for details, Table S5), which were consistent but generally higher than employee shift exposures, reflecting the time spent by employees in less smoky break rooms and offices in addition to the main casino floor. The maximum 1-min exposures for visitors in smoking slot areas (grouped) ranged from 136 to 411 µg/m^3^, reflective of intermittent time spent close to other patrons. The visitor exposures in nonsmoking areas and the outdoors were much lower than in the smoking slot areas, except for Days 3 and 4 during which wildfires in the region resulted in high outdoor PM_2.5_ levels. Outdoor PM_2.5_ penetrating the nonsmoking area to a greater degree than the smoking areas could explain this result, e.g., if air came in directly through the event center doors or the nearby doorway to the outdoors, whereas outdoor air entering the smoking areas mostly passed through the HVAC filters. On days without active wildfires, the daily aggregate nonsmoking area exposures had means of 4.3 to 13 µg/m^3^, and the outdoor exposures were 0.43 to 7.6 µg/m^3^. On days with wildfires, the exposures in smoking areas were similar to those on days without fires. These results indicate that indoor PM_2.5_ levels in smoking areas, even on days with wildfires, are almost entirely due to smoking.

Our data on mean employee exposures are consistent with the values of 22 to 140 µg/m^3^ that Achutan *et al.* [8] found, and our mean visitor (patron) exposures in smoking areas are in the middle range of the values of 18–183 µg/m^3^ (mean of 63 µg/m^3^) reported by Jiang *et al.* [5] for comparable visits to 36 Indian casinos in California. Our data on maximum PM_2.5_ exposures at Win-River are some of the first reported for a casino environment. Jiang *et al.* [5] report broad ranges of 1-min maximum levels of 44 to 291 µg/m^3^ (average of 116 µg/m^3^) for visiting patrons in California casinos, which are comparable to what we found at Win-River. Maximum exposures are relevant for acute health effects, including respiratory, sensory and irritation effects such as coughing, wheezing, runny eyes, or onset of asthma attacks. Junker *et al.* [32] reported a median level for onset of irritation after brief exposures to SHS particles of 4.4 μg/m^3^; the 1-min levels to which Win-River workers were exposed prior to the 100% smoke-free policy were over 20 times higher.

Table 1 highlights the PM_2.5_ pre-post policy monitoring results over the Friday and Saturday evenings in 2011 (pre smoke-free policy) and 2014 (post smoke-free policy). These results reflect grouped data for between one and three investigators on each evening. 

**Table 1:** Mean and maximum PM_2.5_ [μg/m^3^] from 1-min concentrations + grouped time (*HH:MM*) visitor personal-exposure measurements in four Win-River Casino locations before and after a 100% Casino-wide smoke-free policy was adopted ^**a**^.

Location	Statistic	Before Smoke-Free Policy Adoption 2011	After 100% Smoke-Free Policy Adoption 2014	Change [%]
Outdoors ^**b**^	Mean	3.1	11	+255%
	Max	12	62	+417%
	*Grouped Time*	0:59	2:25	
Main Slots ^**c**^	Mean	72	1.1	−98%
	Max	147	14	−90%
	*Grouped Time*	0:58	3:43	
Corner Slots ^**d**^	Mean	54	1.1	−98%
	Max	104	5.6	−95%
	*Grouped Time*	0:37	1:31	
Nonsmoking ^**e**^	Mean	5.5	2.7	−51%
	Max	15	9.3	−38%
	*Grouped Time*	0:59	1:15	

^**a**^ Data in the table were calculated for Friday + Saturday and 1–3 investigators for each of the before and after monitoring periods (2 days per year) using 1-min SidePak AM510 readings that were mass-calibrated for SHS (see text for more information). The grouped time is the total time spent in a given location by ALL visitors, over which the overall average and maximum values were determined. For both the before and the after measurement periods, data were measured on Friday and Saturday evenings between 8 pm and midnight. In 2011, 46 and 63 active smokers were observed inside the smoking slots and table areas of the casino on Friday night (two counts between 10:45 pm and 11:30 pm) and 36 and 40 on Saturday night (same time frame). These corresponded to 9% to 13% of the total number of patrons in these areas. In 2014 no active tobacco smokers were observed inside the casino during the measurement period, although some patrons were observed using e-cigarettes; ^**b**^ Readings taken outside the main (front) casino entrance; ^**c**^ Readings taken in the main area with slots machines in the center of the casino; ^**d**^ Readings taken in the slot area in a corner of the casino but still connected to the main area; ^**e**^ Readings taken in an area separate from the main and corner slot areas with a sliding door. In 2011, the “Nonsmoking” area was the only area of the casino where smoking was prohibited. In the 2014 visit, we still refer to this area as “Nonsmoking” even though smoking was not allowed in any indoor area of the casino, including the former Nonsmoking area and all other areas.

After smoking was restricted everywhere in the casino, mean levels dropped by 98% in the main and corner slots area where smoking was allowed and by 51% in the designated 2011 Nonsmoking area. This result is similar to that found by Marin *et al.* [33] after smoking bans in San Juan, Puerto Rico, casinos resulted in average PM_2.5_ levels in 10 San Juan metropolitan area casinos decreasing by 88.5%. In Win-River, maximum (1-min) concentrations in smoking indoor locations decreased by 90% or more. Mean outdoor levels increased by over 250%, and maximum 1-min outdoor levels increased by over 400%, which appears to be due to the presence of many patrons smoking outdoors immediately near the Casino’s front entrance. 

Indoors, we observed 46 and 63 active smokers (two repeated counts between 10:45 pm and 11:30 pm) on the Friday evening in 2011 and 36 and 40 counts on Saturday for a similar time frame. Based on active and total patron counts, we found that between 9% and 13% of patrons were observed smoking inside during the 2011 visits. We did not observe any active indoor tobacco smokers in the 2014 visits, although a few electronic cigarette smokers were present. 

**Figure 3:**
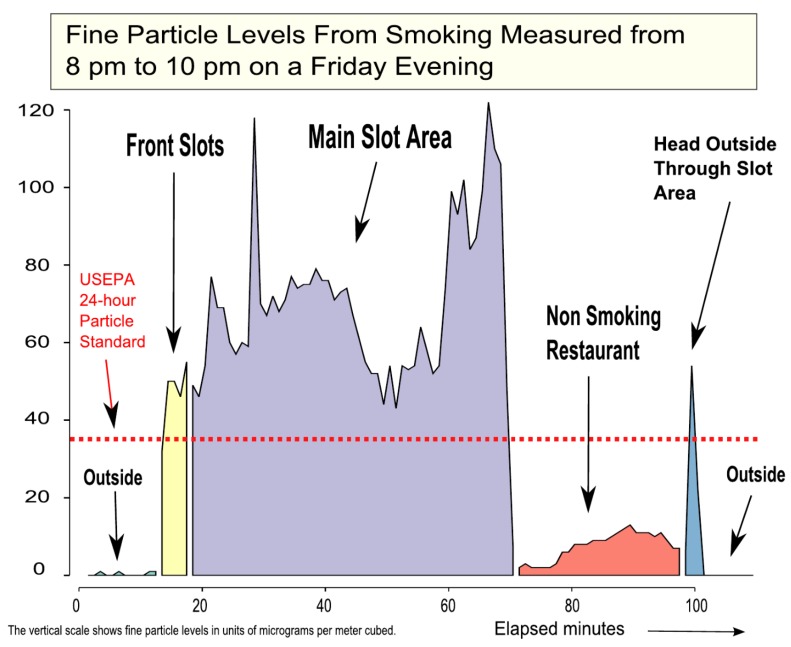
Sample presentation plot showing real-time 1-min fine particle (personal exposure) concentrations (PM_2.5_) measured in the casino by a CAC visitor acting as a patron on a Friday evening in May 2008. This chart was constructed to be accessible to laypersons and is similar to one that was included in the slide presentation by casino General Manager Gary Hayward to Redding Rancheria Tribal Membership and Tribal Council in August 2008. The U.S. EPA 24-h health-based-standard for fine particles of 35 µg/m^3^ is shown as a red horizontal dotted line.

**Figure 4:**
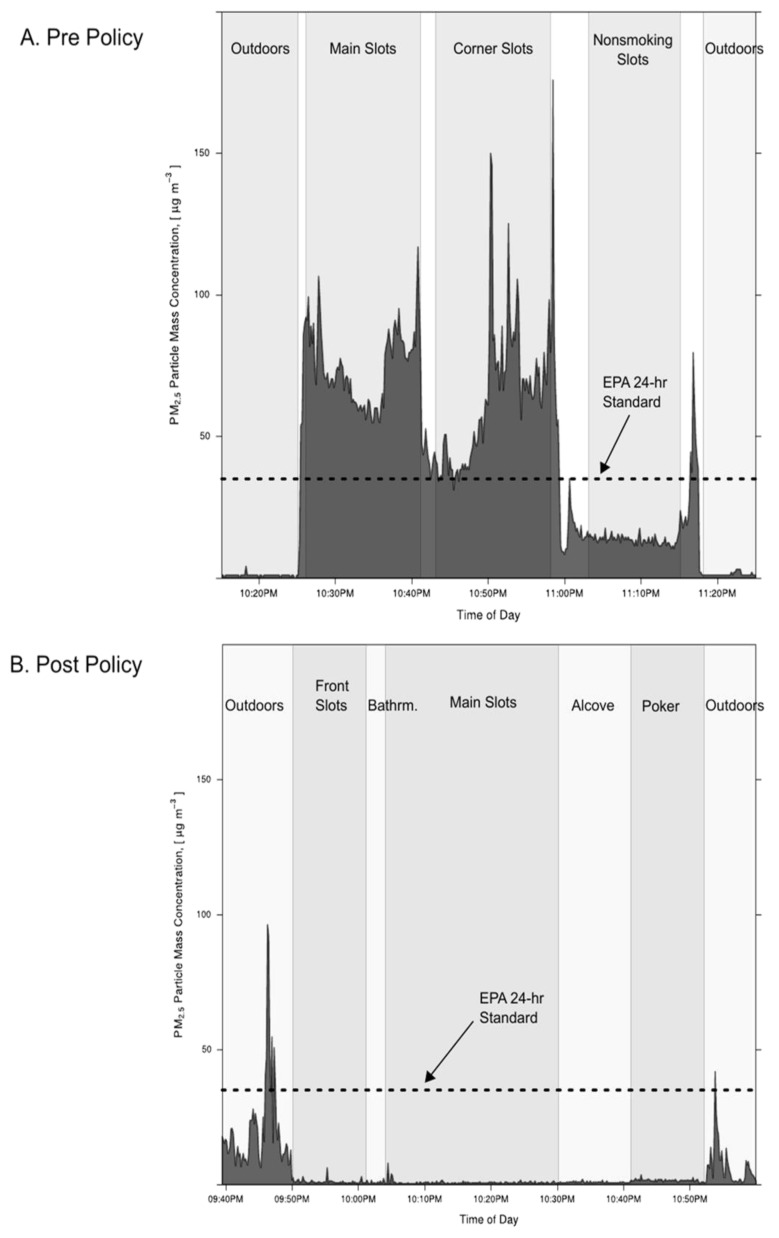
Plots illustrating the change in real-time 10-s fine particle (personal exposure) concentrations (PM_2.5_) from before to after the adoption of a 100% casino-wide, smoke-free policy as measured by CAC visitors acting as patrons (sitting at slot and table games). The reduction in indoor particle levels after the smoke-free policy went into effect is apparent, as well as the increase in outdoor levels: (**A**) a Friday night in 2011 before the casino-wide smoke-free policy was adopted; and (**B**) a Friday night in 2014 after the smoke-free policy was adopted. The U.S. EPA 24-h health-based-standard for fine particles of 35 µg/m^3^ is shown as a horizontal dotted line. Approximately 40–60 active smokers were observed inside the ~20,000 square-foot smoking-allowed (slots + tables) area of the casino during the monitoring period in 2011. No tobacco smokers were observed inside the casino during the 2014 monitoring period, although several active e-cigarettes were observed.

As examples of measured real-time PM_2.5_ personal exposure profiles, Figure 3 (“layperson-friendly” version presented to tribal membership) and Figure 4A (top panel) show profiles measured by a CAC investigator acting as a typical patron in 2008 and 2011, respectively, when smoking was still occurring in the casino. Figure 4B (bottom panel) shows a profile of likely patron exposure after the casino 100% smoke-free policy was put into effect in 2014. The U.S. EPA 24-h PM_2.5_ standard is shown as a dotted line for reference. These plots clearly illustrate a decrease in PM_2.5_ exposure for patrons visiting the smoking-allowed casino versus the 100% smoke-free casino. Although the U.S. EPA does not consider the 35 µg/m^3^ standard to be exceeded unless the average over 24 h exceeds 35 µg/m^3^, the standard is a useful benchmark for making air quality comparisons. When smoking was still allowed in 2011, the levels in the “main” and “corner” slot areas were substantially above this U.S. EPA benchmark, with some peaks above 100 µg/m^3^, compared with very low levels measured outdoors (under 5 µg/m^3^). In contrast, after the smoke-free policy was instituted in 2014, the levels in the main slot areas were very low (under 5 µg/m^3^), while the outdoor levels were higher. All the indoor levels in 2014 were much lower than this U.S. EPA benchmark. The elevated outdoor levels in 2014, which sometimes exceeded 25 to 50 µg/m^3^ and were distinctly higher than indoor PM_2.5_, were measured in the presence of a number of observed active smokers at the front casino entrance. These outdoor PM_2.5_ levels are similar to SHS levels observed by Klepeis *et al.* [34] in various outdoor locations. Some of the outdoor smoke at the casino entrance may have entered the casino in the immediate area of the front slots near the front entrance.

Prior work suggests that the predominant aerosol in smoking-allowed casinos is from tobacco smoke emissions [5,6,7]. Based on the results in Figure 4 and Table 1, our results provide further strong evidence to support this finding. The agreement between the controlled laboratory tobacco calibration factors and the on-site casino calibration factors support the conclusion that nearly all the aerosol is from tobacco smoke. Furthermore, the PM_2.5_ levels before the 100% smoke-free policy were substantial, with means in smoking areas up to 80 or 90 µg/m^3^, which could potentially cause adverse acute and chronic health impacts. Patrons or staff who spend many hours in the smoky air of the casino could receive an exposure that exceeds the U.S. EPA 24-h 35 µg/m^3^ health-based PM_2.5_ standard with momentary levels sometimes exceeding 100 µg/m^3^. In contrast, after the 100% smoke-free policy was adopted, the PM_2.5_ levels were very low and comparable to clean outdoor levels—dropping by effectively 100% and reducing risks to patron and employee health. Our results highlight an important finding that state-of-the-art ventilation and particle-filtering equipment cannot be expected to adequately control SHS exposure, even at ventilation rates exceeding average residential values by many times. The high-powered HVAC system was not sufficient to prevent substantial SHS exposure from occurring in the Win-River casino prior to the institution of the 100% smoke-free policy.

### 3.2. Stakeholder Responses

#### 3.2.1. Initial Survey Responses—2008

Of 297 casino patrons who responded, 49.7% reported being current smokers, and 98.6% of these smoking patrons reported smoking while they play. However, 81.1% of nonsmoking patrons said smoke in the casino bothered them. Reflecting upon potential policy changes, 56.9% of patrons said they would come about the same or more often to Win-River if the casino were to implement a No Smoking policy. However, 43.1% said they would come less often or never if the casino were to go smoke-free. Among nonsmoking patrons, 96.6% said that they would come about the same or more often if the casino were to implement a No Smoking policy. These results are similar to those reported by Brokenleg *et al.* [35] for the Lake of the Torches Resort Casino in Lac du Flambeau, Wisconsin, in which 520 of 957 or 54% of surveyed patrons (sampled from the players club) said they would be more likely to visit more if the casino prohibited smoking. Timberlake *et al.* [36] report, based on the 2008 California Tobacco Survey, that 97% of never-smoker patrons would be more likely to visit a smoke-free casino, and only 23% of current smokers would be less likely to visit.

The results of the casino employee survey indicated that 28.3% of the 153 casino employees who responded reported being current smokers. At a time of limited smoke-free area coverage, 36.9% of the smoking employees said they were “very often” bothered by smoke in their work area, and 71.1% said they were “sometimes” or “very often” bothered by cigarette smoke. By contrast, 86.6% of nonsmoking employees said they were “sometimes” or “very often” bothered by cigarette smoke. Overall, 56.2% of the employees said they preferred to work in a nonsmoking casino. 82.3% either preferred working in a nonsmoking casino or had no opinion. Among non-smokers, 67% preferred to work in a nonsmoking casino, while another 22% had no opinion.

In response to questions about a smoke-free policy, 87.7% of 57 Rancheria employees who responded said that they either would support a No Smoking policy at Win-River Casino or had no opinion on the matter. 12.3% were opposed to the potential policy. Among non-smokers, 85.4% supported a No Smoking policy, while another 6.3% had no opinion on the matter. 75% of Rancheria member respondents said that they either would support a No Smoking policy at Win-River Casino or had no opinion. 25% were opposed to the potential policy (Note: These opinions were gathered in early summer 2008 prior to any knowledge of the results of air quality monitoring, which was presented in August 2008).

In summary, across all four groups, 41.6% of the 519 respondents to the surveys showed direct support, while 68.1% either directly supported the policy or had no opinion. 31.9% were in direct opposition to the policy. These results show that, in spring and early summer 2008, there was generally already some support for a casino-wide smoke-free policy (prior to information on site-specific SHS levels being presented to the tribal membership).

#### 3.2.2. Follow-Up Survey Responses—2012

The overwhelming majority of patron respondents (90%) indicated that they frequented Win-River Casino “weekly”. Of the 45% of patrons who indicated that they currently smoke, almost all (99%) said they usually smoked while playing at the casino. Almost all nonsmoking patrons (95%) said they would visit the casino “more often (43%) or about the same (52%)” if the casino were to prohibit indoor smoking. Only 24% of the smoking patrons indicated they would visit the casino “more often (1%) or about the same (23%)”. The qualitative comments contained a slightly higher proportion of support for a nonsmoking policy (43%) compared to support for the *status quo* (37%). This outcome may be due to the slightly higher percent of respondents who indicated they were non-smokers (55%). 79% of casino employees indicated they were not current smokers. 60% of the employees indicated patrons smoked “very often” or “sometimes” in their area of work. 71% indicated they were “very often” or “sometimes” bothered by patrons smoking in their area of work. The majority of employee respondents (59%) indicated they would prefer to work in a nonsmoking section of the casino, and 53% would prefer to work in a casino that is entirely smoke-free. Among the qualitative comments from employee respondents, support for the casino going smoke-free arose primarily from: (1) concerns of SHS exposure to health; (2) frustrations with the existing ventilation system; and (3) a belief that going smoke-free would increase the number of patrons to Win-River. On the other hand, lack of support for a smoke-free policy was expressed as a fear of losing patrons, and, therefore, facing job layoffs, and a belief that a larger nonsmoking section or simply updating the air filtration system would be sufficient.

#### 3.2.3. Focus Group Results—2013

Participants in the patron focus groups narrowed down a list of the top benefits and barriers to smoke-free policy. Some benefits they identified included a cleaner, non-smoky environment, clothes not smelling like smoke after a casino visit, increase in business, not getting sick as easily, increased revenue for the community, and reduced danger when patrons using oxygen tanks are inside the casino. The top-ranked benefit for responses combined across all three focus groups was a belief that there would be an increase in revenue after a nonsmoking policy, followed by the benefit of a non-smoky environment. Example quotes include: “I won’t get sick so quickly, (cold, runny eyes, nose)”, “More people will come“, “Increase in business”, “I won’t have to breathe in other people’s smoke”, “Cleaner”, and “My clothes won’t smell when I go home”. Barriers brought up by respondents included potential loss of business to neighboring casinos, loss of jobs for employees, the feeling that the issue of health was not a priority for the Tribal Council, and the backlash from unhappy patrons who are smokers. The top-ranked barriers were the potential loss of business, followed by the feeling that tribes do not care about patrons’ health, and a potential loss of jobs for employees. These results suggest that the financial health of a casino after the adoption of a nonsmoking policy holds the key for these stakeholders, since an increase or decrease in revenue was identified as both the top-ranked benefit and barrier.

#### 3.2.4. Town Hall Meeting and Feedback Cards—2013

Results from the patron Town Hall meeting and feedback cards echoed much of what had been identified through the focus groups and surveys. Supporters of a No Smoking policy expressed that a smoke-free environment would lead to better health for all, including casino patrons, restaurant patrons, and casino employees. Other supporting reasons included an increase in business, since a smoke-free environment would attract more non-smokers and families; respondents also noted that the casino would simply be following established California state laws that outlaw smoking in public places. Respondents who preferred that the casino continue to allow smoking asserted that the casino would lose business and patrons if it went smoke-free. Patrons who identified themselves as smokers warned that they would leave the casino for a different casino that allowed smoking. They also expressed that a casino was one of the last few public places that allowed smoking outside of their own homes, and they were unwilling to lose that privilege.

#### 3.2.5. Employee Post-Policy Survey Responses—2014

About half (52%) of the 2014 Win-River Organizational Climate Survey employee respondents (*n* = 241) indicated that they had worked at Win-River for less than 5 years; another quarter had worked there between 5 and 10 years. About 66% of respondents worked in facilities, security, gaming, cage & count, or food and beverage departments. Essentially all of the respondents (99.6%) worked 8 or more hours a day, with 54% previously working in a smoking area (before the 100% smoke-free policy). Half of the respondents were non-smokers, and another 28% previously smoked but have since quit. A large majority of respondents (87%) lived in a smoke-free home. Of current smokers (*n* = 76), close to two-thirds (or 48 respondents) indicated that they were now “much more likely or somewhat more likely” to quit smoking due to the implementation of the smoke-free policy. Some respondents gave specific comments on health improvements or improvements in work environment after the smoke-free policy (*n* = 141 and 147, respectively). They generally commented that they experienced less coughing, breathed better, and suffered less irritation to their nose and throat after the casino went smoke-free. They generally reported that the casino didn't smell, was cleaner, and people were happier. Of the 129 respondents who gave open-ended comments on the smoke-free policy, ~90% were supportive with statements such as “great job”, “I love the smoke-free policy”, “it is awesome”, and “it is great for people in general”. These results show that a smoke-free environmental policy is, in general, highly-valued by many casino employees, with approximately one-third who took the time to fill out the survey and answer the final open-ended comments voicing strong support for the adopted smoke-free policy. 

### 3.3. Tribal Leadership Decision Making

According to reports from General Manager Gary Hayward, the fine-particle (PM_2.5_) information, together with the smoker-count surveys, and the patron, employee, and tribal council surveys, were crucial to the Tribal Council’s confirming decision to implement the 100% smoke-free policy at the Win-River Resort and casino. The focus group and Town Hall meetings had a lesser, but still contributing role. The data were formulated in layperson’s terms (with clear graphics) to enable the casino general manager to clearly relay findings to tribal council members and ensure that the issue did not fade due to lack of education and information. The CAC technical assistance provided input into the casino's internal cost-benefit analysis related to maintenance or refurbishing costs due to smoking and potential health effects for employees who are exposed to SHS.

Manager Hayward shared all information from stakeholder activities—as it became available—with the Tribal Council at the regularly-scheduled monthly meetings between 2008 and 2013. Data from the air quality results and results of initial 2008 employee (casino and Rancheria), patron, and council surveys were presented to the General Membership in August 2008. Figure 3 is an example of a “layperson-friendly” air quality chart that was used in the presentation, showing the real-time PM_2.5_ exposure profile of a casino visitor. Information on airborne nicotine and PM_2.5_ fixed-site concentrations, employee and visitor average and peak PM_2.5_ exposures, and visitor cotinine levels (*ca.* 2008), similar to those included in the Supplement (Table S2, Table S3, Table S4 and Table S5), were also presented to the membership. Following the presentation, the General Membership were 100% in favor of Manager Hayward gathering information for a potential move to a smoke-free policy. Initially, the Tribal Council was roughly evenly split on the issue of a new smoke-free policy. As more data came in over subsequent years and the council changed members, it evolved into a 70%–30% split in favor of the policy from about 2010 onward, and it ended up with 80% in favor of the policy in January 2014 when the council voted to move forward with the smoke-free policy.

#### 3.3.1. Content of August 2008 Slide Presentation

The slide presentation made by Manager Hayward to tribal members in August 2008, with key input from Ms. Dhaliwal and other CAC members, contained critical information on air quality and employee and patron opinions that swayed the General Membership to be 100% in favor of moving toward a smoke-free policy. This presentation contained a variety of details on Win-River-specific SHS exposures for employees and patrons and opinion surveys. The key elements of the presentation are summarized in Table 2.

**Table 2:** Elements of slide presentation by general manager to tribal membership.

Slide Element	Description
Constituents of SHS	4000+ Chemicals; Carcinogens
Health Effects of SHS	California Air Resources Board and Surgeon General deem SHS a Toxic Air Contaminant with “no safe level of exposure,” respectively
External Surveys	Shasta County Residents support smoke-free casinos; California Indian Health Board Survey shows 80% of patrons + staff prefer a smoke-free environment and casino gamblers cited “too smoky” as #2 reason to not go more often; J.D. Power and Associates report that the overwhelming majority of gaming customers desire a smoke-free environment
Internal Surveys	A majority of Win-River gamblers would come more often with a smoke-free policy; majority of casino employees would like to work in a smoke-free environment, and a large majority of Rancheria employees support a smoke-free policy
Air Quality Evaluation at Win-River	Charts show real-time exposure visitor profiles; nicotine detected; elevated particle levels; personal exposure to short, very high levels; rise in visitor cotinine indicates significant SHS exposure
Business Costs	U.S. Surgeon General concludes smoke-free policies have no negative impact on revenue; savings due to reduction in morbidity; lower insurance rates
Non-Tribal Casino News	Las Vegas poker rooms are smoke-free; casinos and card rooms in California are 100% smoke-free; Colorado and New Jersey passed smoke-free laws
Tribal Casino Steps	Casinos moving toward restrictions on smoking; 3 casinos have gone smoke-free with no loss in revenue—Blackfeet, Taos, Lucky Bear

#### 3.3.2. Perceived Barriers and Key Factors for a 100% Smoke-Free Policy

As reported by Manager Hayward in his key-informant interview based on tribal meetings, a main barrier to smoke-free policy adoption by the Tribal Council was the responsibility of the Redding Rancheria to provide economic income for a large tribal population. The casino is the main economic driver for the Tribe, supplying funding for tribal programs (education, health care) and individual members. Employee health wasn’t considered as much of an issue initially, but it grew in influence as more information came in.

Manager Hayward argued to the Tribal Council and General Membership that adding the hotel would bring about an overall increase in income, so it might be a good time to try the smoke-free policy since any potential loss in revenue would be compensated by the hotel income. While all of the General Membership who attended the August 2008 meeting were 100% supportive of investigating a smoke-free policy, the Tribal Council members, who make the final vote on policies, were concerned about revenue, in addition to a healthier workplace. However, the members of the Tribal Council decided that the prospective hotel revenue increases would likely offset any initial possible revenue decrease due to the establishment of a comprehensive smoke-free policy.

In a formal key informant interview with an alternate Tribal Council member on 11 February 2015, just after the vote to roll back the smoke-free decision, he stated that the “Tribal council were split on the [100%] implementation. Half of them are smokers. When passed, majority wanted it with support of general membership.” When asked about the reasons that any members of the council opposed the initial 100% smoke-free policy, he stated “None, other than a couple smokers on tribal council.”

Based on Manager Hayward’s interview results on tribal meetings, of all the information communicated to the council and membership, the simplified visual charts of material had the most impact on the opinions of casino membership and council, since they were the easiest to understand. The data from the air quality measurements raised awareness, in part, because the approach was novel for most casino members, and the results broken down by casino area gave clearly-understandable comparisons (*i.e.*, smoking area versus nonsmoking area versus outdoors). The active-smoker counts clearly showed how a small percentage of people smoking could have a large effect on overall air quality. When asked specifically in his interview “Which pieces of data were more crucial?” when recalling events after the smoke-free policy was implemented, Mr. Hayward responded: “Definitely the air monitoring, the level of exposure data—that’s definitely what people would have to think about—long term.” 

According to Manager Hayward’s feedback to the CAC, the survey opinion data had an impact on the tribal council and membership, but it was not as large a deciding factor as the air quality data. The survey data may have had too many nuanced responses per question to provide a clear picture. In addition, having more survey data from the surrounding area where residents may patronize the casino (Shasta, Trinity, and Butte counties) may have helped. In comparison to the surveys, the focus group results had little impact on council opinions, since they were not perceived as involving a big enough test group.

#### 3.3.3. Roll-Back to a 70% Smoke-Free Policy

In February 2015, the Redding Rancheria Tribal Council voted to amend the Win-River casino-wide smoking restriction to a restriction on 70% of the casino floor. In general, we expect that the adoption and expansion of smoke-free policies by casinos are inherently dynamic, which may be due to shifts in business models that focus on a gaming and resort destination [37]. Specific to Win-River, late in 2014 the casino experienced a reduction in revenue (after an initial increase in revenue just after the smoke-free policy went into effect) and an increase in complaints from the smoking-minority patrons. Our work with the Win-River casino towards maintaining a 100% smoke-free policy continues to the present.

A confluence of factors may have contributed to reduced casino patronage and revenue, including poor weather, lower gasoline prices (facilitating longer drives) and more aggressive marketing to local fixed-income patrons by casinos that still allow smoking in nearby communities. Revenue decreases started to affect membership dividends, as well as employee staffing hours and morale. Consequently, the Tribal Council became split over the smoke-free issue amid concerns of maintaining income—with a slight majority in favor of retreating from a 100% smoke-free policy. 

While we do not have access to precise individual Tribal Council member opinions, responses from recent interviews with Manager Hayward and a former alternate council member offer insight into deliberations at tribal meetings. Mr. Hayward stressed the challenges of maintaining a smoke-free policy that coincides with lost revenue: “...it’s a big issue when the tribal members rely on that revenue to survive...”. “[Casinos] need to consider whether they can sustain [revenue loss] for a long period of time —what would be a sustainable reduction in their revenue for what period of time?” It was clear to staff that patrons who smoke were lost to other casinos and that smokers contributed a high proportion of income for Win-River. One way to mitigate revenue losses may be to join forces with nearby casinos, as Mr. Hayward suggests: “[Competitors] started taking an interest but they also exploited it. But all of this could have been deterred if we had formed a coalition....If the customer didn’t have anywhere else to go, they would have to make [an] adjustment.” “We found out that...20% of our patrons make up 80% of our revenue. And it turned out that the 20% were smokers. And these people came back—you can see it—it’s a direct correlation with that. We didn’t increase our promotions after reversal—no extra incentives. But they came back [after the policy reversal].”

The alternate council member stated in his interview that: “[It was] sad for employees and patrons who do not smoke, the decision to reverse was unexpected really, and decision was to give it one year. I don’t think it has been given long enough and given time would show an increase in revenue I feel. Keep fighting the battle, it’s a good cause as a former smoker from way back when...Membership was divided on going back to allowing smoking, council vote only went through by one vote. General membership split. Just a bump in the road!”

## 4. Conclusions and Recommendations

Tribal-owned-and-operated casinos in California and elsewhere that allow smoking indoors put patrons and staff of the casino at risk to high secondhand tobacco smoke (SHS) exposure and resulting acute or chronic health effects. As demonstrated in the present exploratory effort, measurement of air pollutant exposures associated with smoking in casinos can provide clear real-time data on contamination and convincing evidence that 100% smoke-free policies protect patrons and staff. Providing scientifically credible and detailed air monitoring, which can stand up to scrutiny in the scientific community, is an important element to allow Tribal Council members to proceed with confidence and clarity. Clearly, from our results, the high SHS exposure levels and resulting health risks associated with many active indoor smokers at Win-River casino were no longer present after the 100% smoke-free policy went into effect, providing conclusive evidence that a small number of actively-smoking patrons caused essentially all of the PM_2.5_ measured before the smoke-free policy. The Casino’s strong ventilation and filtration system was inadequate to protect workers and patrons from high levels of SHS exposure when smoking was allowed. Our methods and findings proved useful and influential in the Redding Rancheria Tribal Council deliberations, and may provide valuable data for smoke-free efforts at other casinos.

Six factors should be considered in future intervention studies and public health initiatives to promote smoke-free casinos. Based on our experience at Win-River, the three essential factors are: (1) Having at least one smoke-free champion among casino management who leads communication of technical and other supportive information to decision makers; (2) Availability of scientifically credible, site-specific assessment and clear presentation of air quality and health risk impacts of SHS; and (3) Having dedicated public health and environmental health researchers and scientists closely involved in an advisory role. The concerns over health and contamination are important for securing support of most tribal members. However, in the case of Win-River, we found that revenue concerns ultimately overwhelmed health concerns, leading to policy reversal. Casinos may consider addressing revenue concerns systematically in three interacting ways: (1) Understanding the true nature of income from smokers by obtaining survey information on smoking status of “players” and, critically, how much they play (how much revenue they actually generate); (2) Obtaining buy-in from other casinos, possibly forming a coalition that seeks coordinated smoke-free policies, thus avoiding loss of smoking patrons to competing casinos; and (3) Given that adoption of a 100% smoke-free casino may coincide with some initial revenue loss, management should clearly identify the level and period of revenue loss that can be sustained by the tribe. 

This unique, exploratory collaboration illustrates how providing feedback to casino management, and the tribal councils that oversee them, on potential SHS exposure and survey results can lead to policies that eliminate or substantially reduce SHS exposure and health risks. Our approach and findings may provide valuable background data and templates or guidelines for other public health workers and casino managements seeking restrictive smoking policies. The scientific and public health benefits to reporting on the before-and-after air pollutant concentrations in the casino include an improved understanding of the contribution of smoking to indoor air pollution in gaming and hospitality establishments with evidence of the impact of smoking on employee and patron health risks. Our work with Win-River provides a model and motivating force for future casino interventions and smoke-free policies. Furthermore, our data on SHS employee exposure may be useful as part of quantitative risk and cost assessments associated with smoking in casinos.

## Supplementary

**Table S1:** Historical time-line of Win-River and smoke-free policy-consideration activities.

Date	Activity
1987	Redding Rancheria Health Clinic established
1993	Redding Rancheria Win-River bingo hall established
1999	Initially approached by Shasta County Local Lead Agency for tobacco control
1999	Unsuccessful bid for smoke-free Win-River casino
1999	Implementation of a smoke-free area in casino
May2008	Gary Hayward (Redding Rancheria and Win-River) reaches out to Shasta County Tobacco Education Program (SCTEP) for technical assistance on SHS information
May 2008	Casino Advisory Committee (CAC) formed consisting of SCTEP, California Clean Air Project (CCAP), and environmental health scientist Dr. Neil Klepeis
May/June 2008	Environmental monitoring of SHS exposures in the casino (airborne nicotine, PM_2.5_, urinary cotinine)
June/July 2008	Opinion survey mailed to 1000 patrons (297 returned) on adoption of a smoke-free casino policy ($10 free-play incentive); 153 casino employees, 57 Rancheria employees, and 12 Tribal members also responded to separate surveys. (Survey respondents were not aware of air quality monitoring results)
August 2008	Slides adapted and presented by General Manager (Gary Hayward) to Tribal Council and Tribal Membership. Tribal General Membership votes to research the adoption of a smoke-free policy (explore the issue); gives direction to pursue more research (100% vote yes)
2 December 2008	Gary Hayward presents talk on Win-River experience at *Smoke-Free California Conference*
February 2009	Decision to implement a smoke-free policy delayed due to financial downturn; construction stalled
2008–2010	CAC meets with casino management 6 times, with individual meetings between Ms. Dhaliwal and Gary Hayward ~8 additional times (plus many email, text, and phone conversations) providing tobacco education materials and preparing a slide show on SHS, survey results, and air monitoring results
July 2010–July 2013	CAC has continuous contact with Win-River & Redding Rancheria by CCAP to maintain the smoke-free issue; Technical assistance provided in-person, by phone or email
December 2011	CAC performs follow-up air-monitoring survey of fine particles (PM_2.5_) in the casino
June 2012	CAC mails follow-up opinion surveys to 1990 casino patrons (1250 returned; $10 free-play incentive); 97 casino employees also responded to a separate survey
January 2013	The tribe breaks ground on $90M hotel addition, casino renovation and expansion (bar/cafe)
August 2013	CAC holds two focus groups with 6 casino patrons each
August 2013	CAC holds focus group with local agency staff and members of the community who regularly utilize casino facilities
August 2013	CAC holds Town Hall meeting with 23 casino patrons; air monitoring results presented
December 2013	Tribe completes construction of new smoke-free hotel
23 January 2014	Follow-up information from air monitoring, focus groups, surveys, and Town Hall meeting presented to Tribal Council
23 January 2014	Tribal Council votes to implement smoke-free policy (80% Yes)
14 March 2014	Casino-wide smoke-free policy adopted
26 March 2014	Follow-up PM_2.5_ monitoring after smoke-free policy is put into effect
May/June 2014	Win-River Organizational Climate Survey (with questions on smoke-free policy) distributed to employees
February 2015	Tribal Council votes to roll-back ban to allow smoking on 30% of casino floor; hotel and event center remain smoke-free.

**Table S2:** Urinary cotinine levels in four visitors before and after exposure to secondhand tobacco smoke in the Win-River Casino on a Saturday in 2008 ^**a**^.

Subject	Pre-Exposure Cotinine [µg/L]		^b^ Time Exposed to SHS in Win-River Casino on 6/28 (Sat)	Post-Exposure Cotinine [µg/L]		Change [%]
1	0.29	(6/26 7:27 PM)	3PM–1AM (intermittent)	2.5	(6/28 1:45 AM)	+760%
2	0.35	(6/26 7:00 PM)	3PM–1AM (intermittent)	5.9	(6/28 2:00 AM)	+1586%
3	0.29	(6/27 10:30 AM)	9:30 PM–11:00 PM	2.7	(6/28 1:55 AM)	+831%
4	0.23	(6/27 10:30 AM)	9:30 PM–11:00 PM	9.5	(6/28 3:00 AM)	+4030%

^**a**^ Nicotine levels are the sum of hydroxy cotinine & cotinine and have been normalized for 100 mg/dL creatinine; ^**b**^ Subjects were non-casino-employee visitors associated with the CAC, who reported that they were not exposed to secondhand tobacco smoke (SHS) between the first and second sample other than the time that they were inside the casino. An average of 41 active smokers were observed in the smoking slots and table areas in the evenings of the visit (Day 4) between 9 PM and 11 PM, which was approximately 10% of all patrons.

**Table S3:** Fixed-site airborne nicotine and SidePak-based PM_2.5_ concentrations at different Win-River Casino locations by day of visit ^**a**^.

Day of Visit	Location	Nicotine Time Interval [HH:MM]	Nicotine Mass Collected [µg]	Mean Nicotine Conc. [µg/m^3^]	PM_2.5_ Time Interval [HH:MM]	Mean PM_2.5_ [µg/m^3^]	Max 1-min PM_2.5_ [µg/m^3^]
Day 1 (Fri)	Pit				8:19	65	182
Day 2 (Sat)	Pit				8:31	57	195
Day 3 (Fri)	Stage	8:30	N/A	N/A	8:19	36	71
	Pit	8:35	6	10	8:36	60	110
	Cage 3	8:24	2	4	8:23	39	77
	Cage 2				8:58	61	221
	Cage 1	8:59	3	6	8:57	47	72
	Outdoors				9:18	76	175
Day 4 (Sat)	Stage	10:41	N/A	N/A	10:41	49	178
	Pit	10:07	6	10	10:07	65	172
	Cage 3	10:19	2	3	10:13	52	329
	Cage 2				10:02	70	172
	Cage 1	10:09	4	8	7:15	57	121
	Outdoors				10:03	111	151
Day 5 (Fri)	Stage				8:50	28	55
	Pit	8:31	4	10	8:32	68	192
	Cage 3				8:50	43	72
	Cage 2				8:36	74	183
	Cage 1	8:45	7	8	8:48	42	76
Day 6 (Sat)	Stage				8:55	24	77
	Pit	8:41	7	10	8:43	63	133
	Cage 3				9:06	34	83
	Cage 2	8:17	5	9	6:07	67	134
	Cage 1				8:35	46	156
Day 5+6	Outdoors				32:46	4.8	28
Day 7 (Fri)	Pit				8:05	87	229
Day 8 (Sat)	Pit				9:01	70	206
Day 9 (Fri)	Pit				2:32	0.93	3.6
	Office				2:27	1.0	2.5
	Outdoors				2:25	0.92	28
Day 10 (Sat)	Pit				6:50	1.9	9.8
	Office				6:42	2.2	4.2
	Outdoors				6:40	10	168

^**a**^ Days 1–6 were in May, June, October 2008 (2 days each month), Days 7–8 were in December 2011 and Days 9–10 were in March 2014 (smoke-free policy in effect). Nicotine filter sample pumps and SidePak monitors were started approximately synchronously between 2 PM and 6 PM. Nicotine flow rate was calibrated at ~1 LPM. Blank filters had no detectable nicotine. N/A indicates nicotine samples below the detection limit of 1 µg filter nicotine mass. PM_2.5_ is derived from continuous readings measured by SidePak AM510 Aerosol Monitors calibrated for SHS (see text for more information). Between 30 and 60 active smokers were observed, on average, inside the smoking slots and table areas in the evenings of the 2008 and 2011 visits between 8 PM and midnight. No cigarettes were observed indoors for Days 9 and 10 (2014) although outdoor active-smokers were observed.

**Table S4:** Mean and 1-min maximum personal PM_2.5_ exposures for individual Win-River Casino employees in 2008 and 2011 by day of visit ^**a**^.

Day of Visit	Employee Job	^b^ Shift Monitoring Start Time [HH:MM]	^c^ Time of Sampling [HH:MM]	Mean PM_2.5_ exposure [µg/m^3^]	Max 1-min PM_2.5_ exposure [µg/m^3^]
Day 3 (Fri)	Pit Boss	3:39 PM	6:49	71	539
	Security Guard	2:35 PM	6:42	40	147
	Slot Floor Cashier	4:55 PM	5:31	57	227
	Slot Floor Supervisor	2:35 PM	6:54	46	174
Day 4 (Sat)	Pit Boss	3:48 PM	7:06	71	117
	Security Guard	3:50 PM	7:06	55	119
	Slot Floor Cashier	4:53 PM	5:34	62	305
	Slot Floor Supervisor	3:47 PM	7:04	60	138
Day 5 (Fri)	Pit Boss	6:00 PM	7:01	67	194
	Security Guard	3:05 PM	7:12	55	221
	Security Guard	6:13 PM	5:04	54	701
	Slot Floor Cashier	5:13 PM	4:48	55	164
Day 6 (Sat)	Pit Boss	6:00 PM	6:56	63	354
	Security Guard	3:00 PM	7:05	46	110
	Security Guard	3:00 PM	7:05	39	163
	Slot Floor Cashier	5:02 PM	6:46	51	243
Day 7 (Fri)	Security Guard	3:22 PM	6:05	35	121
	Security Guard	3:22 PM	8:26	39	197
	Player’s Club	3:10 PM	5:43	52	110
Day 8 (Sat)	Security Guard	2:51 PM	6:07	28	99
	Security Guard	2:50 PM	8:57	35	154
	Player’s Club	3:45 PM	5:31	44	129

^**a**^ Days 1–6 were in May, June, and October 2008 (2 days each month) and Days 7–8 were in December 2011. The PM_2.5_ exposure mean and 1-min maximum levels measured during a given employee’s shift are derived from 1-min continuous SidePak AM510 Aerosol Monitor measurements calibrated for SHS (see text for more information). Between 30 and 60 active smokers were observed, on average, in the smoking slots and table areas in the evenings of the visits between 8 PM and midnight; ^**b**^ The start time is the time that the employee arrived and was fitted with a SidePak monitor; ^**c**^ The sampling time did not necessarily correspond to the employees shift duration because the SidePak battery may have been depleted before the end of the shift.

**Table S5:** Mean and maximum 1-min personal PM_2.5_ exposures for visitors in 2008 acting as patrons ^**a**^.

Visit No.	Visitor Location	^b^ Grouped Time [HH:MM]	Mean PM_2.5_ Exposure [µg/m^3^]	Max 1-min PM_2.5_ Exposure [µg/m^3^]
Day 1	Smoking Slots	2:58	73	127
(Fri, 2008)	Nonsmoking Slots	0:28	4.3	41
	Outdoors	1:05	0.43	14
Day 2	Smoking Slots	3:47	82	411
(Sat, 2008)	Nonsmoking Slots	0:29	13	22
	Outdoors	1:08	2.9	5.4
Day 3	Smoking Slots	2:25	63	204
(Fri, 2008)	Nonsmoking Slots	0:15	92	137
	Outdoors	0:53	107	123
Day 4	Smoking Slots	3:10	78	150
(Sat, 2008)	Nonsmoking Slots	0:30	69	125
	Outdoors	1:07	125	137
Day 5	Smoking Slots	5:58	65	200
(Fri, 2008)	Nonsmoking Slots	0:38	7.8	39
	Outdoors	2:06	4.9	14
Day 6	Smoking Slots	4:51	53	136
(Sat, 2008)	Nonsmoking Slots	0:29	8.8	25
	Outdoors	1:09	7.6	14

^**a**^ Days 1–6 were in May, June, and October 2008 (2 days each month). The PM_2.5_ values in the table represent aggregate concentrations for all investigators visiting broad locations in the casino (Smoking or Nonsmoking Slots or Outdoors; Smoking Slots designation includes all main smoking areas with machines in the center, corner, and front areas of the casino—not including the poker area; Nonsmoking Slots designation includes only the nonsmoking slots area separated from the main smoking areas by a sliding door). For each day of monitoring up to 3 investigators performed personal monitoring. Each visitor started their 1 to 2 h personal sampling between 7 PM and 12 PM. Between 30 and 50 active smokers were observed, on average, in the smoking slots and table areas in the evenings of the visits between 8 PM and 12 PM; ^**b**^ The Grouped Time column represents the total time spent by all visitors on a given day (*i.e.*, total averaging time).

## Other Unreported Environmental Measures

In addition to the environmental measures reported in the main article, we also measured levels of CO_2_, temperature, humidity, ultrafine particles, and polycyclic aromatic hydrocarbons using factory-calibrated real-time monitors (Q-TRAK Indoor Air Quality Monitor, Model 7565, TSI, Inc., Shoreiew, MN, USA; Model 3007, Portable Condensation Nuclei Counter, TSI, Inc., Shoreview, MN, USA; Model PAS 3000ce, Ecochem, League City, TX, USA). Some of the on-site gravimetric filters were analyzed by Lawless to estimate the proportion of tobacco aerosol (see citation [38]). However, since the results of these measures were auxiliary or exploratory in nature and were not reported to the Win-River casino management, they are not relevant to the collaboration and we do not report them here.
